# Fibro-osseous pseudotumor of the digit: Case report and surgical experience with extensive digital lesion abutting on neurovascular bundles

**DOI:** 10.1016/j.amsu.2018.09.034

**Published:** 2018-10-04

**Authors:** Tariq Jawadi, Feras AlShomer, Muhammed Al-Motairi, Abdullah Al-Qahtani, Mohammad Alfowzan, Obaid Almeshal

**Affiliations:** Plastic and Reconstructive Surgery, Surgery Department, King Fahad National Guard Hospital, King Abdulaziz Medical City (KAMC), Saudi Arabia

**Keywords:** Fibro-osseous, Pseudotumor, Hand, Pyogenic granuloma, Myositis ossificans

## Abstract

**Background:**

Fibro-osseous pseudotumor (FOPD) of the digit is a rare benign lesion of subcutaneous tissue characterized by fibroblastic proliferation and osteoid formation. Herein, we present a case of massive FOPD lesion in the base of ring finger with extensive involvement of the neurovascular bundles with challenging surgical approach.

**Case description:**

A 27-year old female patient, presented with 7-months history of a progressively enlarging mass on her left hand. Upon assessment, the mass was located over the proximal phalanx of the left ring finger with extensive involvement of the 4th web space. Her neurovascular examination was normal. Radiological investigations showed partial involvement of the radial sided bundle together with complete involvement of the ulnar sided neurovascular bundle. The patient was bothered by the mass being painful with overlying skin ulceration. She was taken afterwards to the operating room where the mass was dissected freely from those bundles while preserving the radial and ulnar structures. The resected margins were however, positive for residual lesions due to the extensive nature of the mass. The patient was informed about the need for close follow-ups for both clinical and radiological signs of lesion recurrence pending early surgical intervention.

**Conclusion:**

FOPD although benign, a soft tissue osteosarcoma is one of the differential diagnosis. Meticulous attention to the clinical, pathological and histological features of FOPD is required. Early diagnosis and treatment of FOPD is very crucial in optimizing the overall outcome. Pre-operative planning with various radiological modalities was of great help anticipating the surgical course.

## Highlights

• Fibro-osseous pseudotumor (FOPD) of the digit is a rare benign lesion of subcutaneous tissue.
• The differential diagnosis includes: myositis ossificans, extraskeletal osteosarcoma, parosteal osteosarcoma.
• Meticulous pre-operative planning is of great importance to optimize patient outcome.
• Fibro-osseous pseudotumor (FOPD) although benign, complete excision assure lack of recurrence.
• Meticulous histological assessment for any features of malignancy assure proper patient outcome.

## 1 Introduction

Fibro-osseous pseudotumor of the digit (FOPD), is a rare benign lesion of subcutaneous tissue characterized by fibroblastic proliferation and osteoid formation [1,2]. Fibro-osseous pseudotumor of the digit was previously prescribed in literature under various names like; florid reactive periostitis, parosteal fasciitis and fasciitis ossificans [1,3]. It is crucial to know the clinical, pathological and histological features of this lesion in order to avoid diagnostic ambiguity. The major differential diagnoses include; myositis ossificans, extraskeletal osteosarcoma, parosteal osteosarcoma and subungal exostosis [1,4,5]. Fibro-osseous pseudotumor of the digit usually presents as a localized proximal nodule that is slightly mobile and affecting more commonly women than men. This lesion is known for its good prognosis if complete surgical excision was attained, with a very low recurrence rate and no reports of malignant transformation [1,4,5].

## 2 Methodology

This is a retrospective chart review and case presentation of a patient presented to our tertiary care hospital. Patient's related history and examination are summarized. This work has been reported in line with the SCARE criteria [6]. The patient was consented for the publication of this work.

## 3 Case presentation

**Fig. 1:**
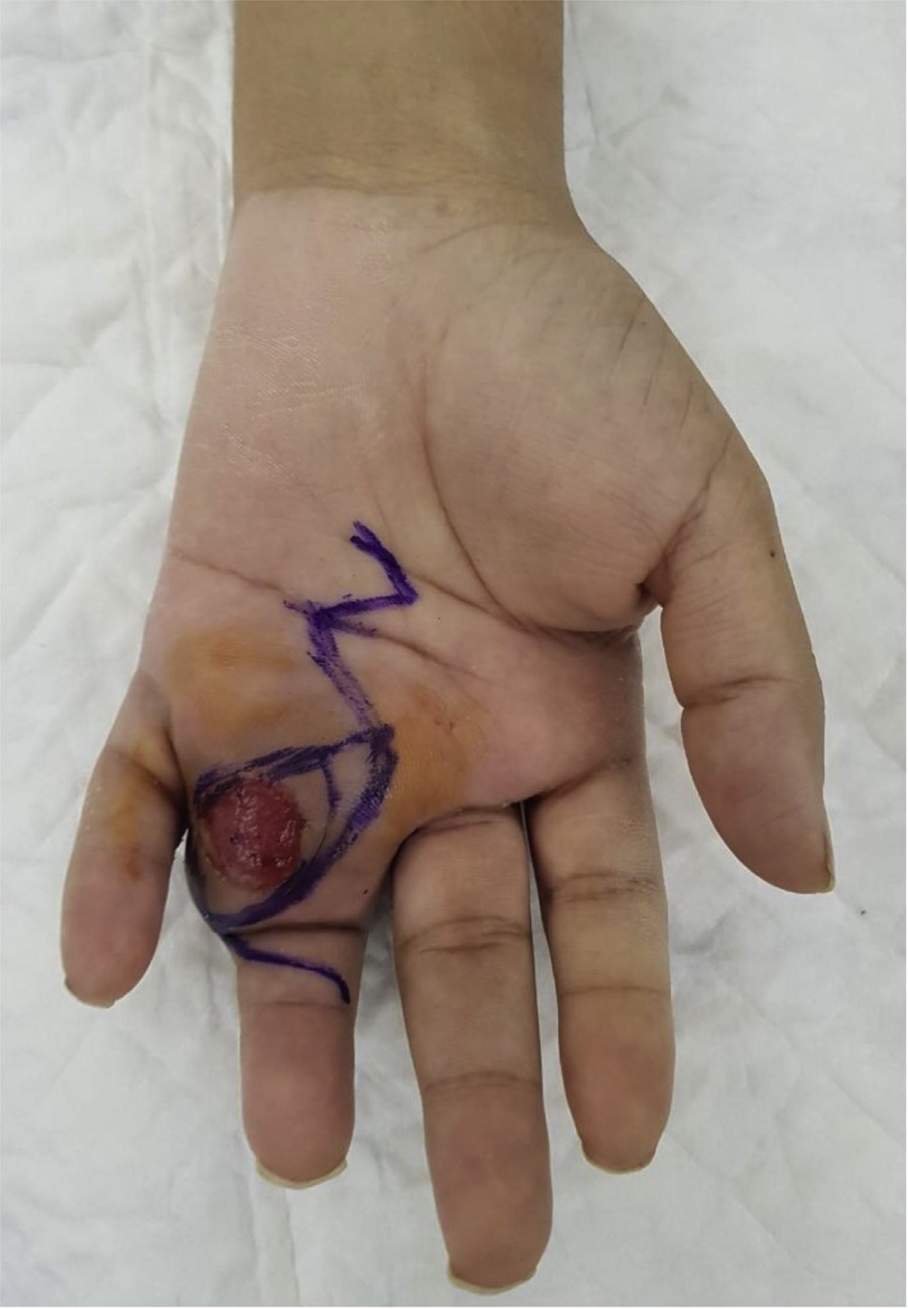
**Clinical presentation of digit Fibro-osseous pseudotumor mass.** It shows the pre-operative clinical presentation of the mass at the base of the left ring finger. Notice the overlying ulcerative skin. Pre-operative incision marking is also shown.

Our patient is a 27-year old female that denied any past medical and surgical history. Her family and drug history were also unremarkable. The patient presented with a 7-month history of progressively enlarging mass on her left ring finger. The patient mentioned that the mass appeared suddenly with no history of trauma and that she was concerned about the potential malignant nature of the mass. The patient also mentioned that she underwent incision and drainage of that mass 2 months after its appearance with no improvement and provided no detailed surgical or pathological reports, which was the reason for her delayed presentation. Upon her assessment, the mass was located over the ulnar side of the proximal phalanx of left ring finger with extensive involvement of the 4th web space. The overlying skin coverage was ulcerative with no active signs of infection. Range of motion of the involved digit was limited, however neurovascular examination was normal. (Fig. 1).

**Fig. 2:**
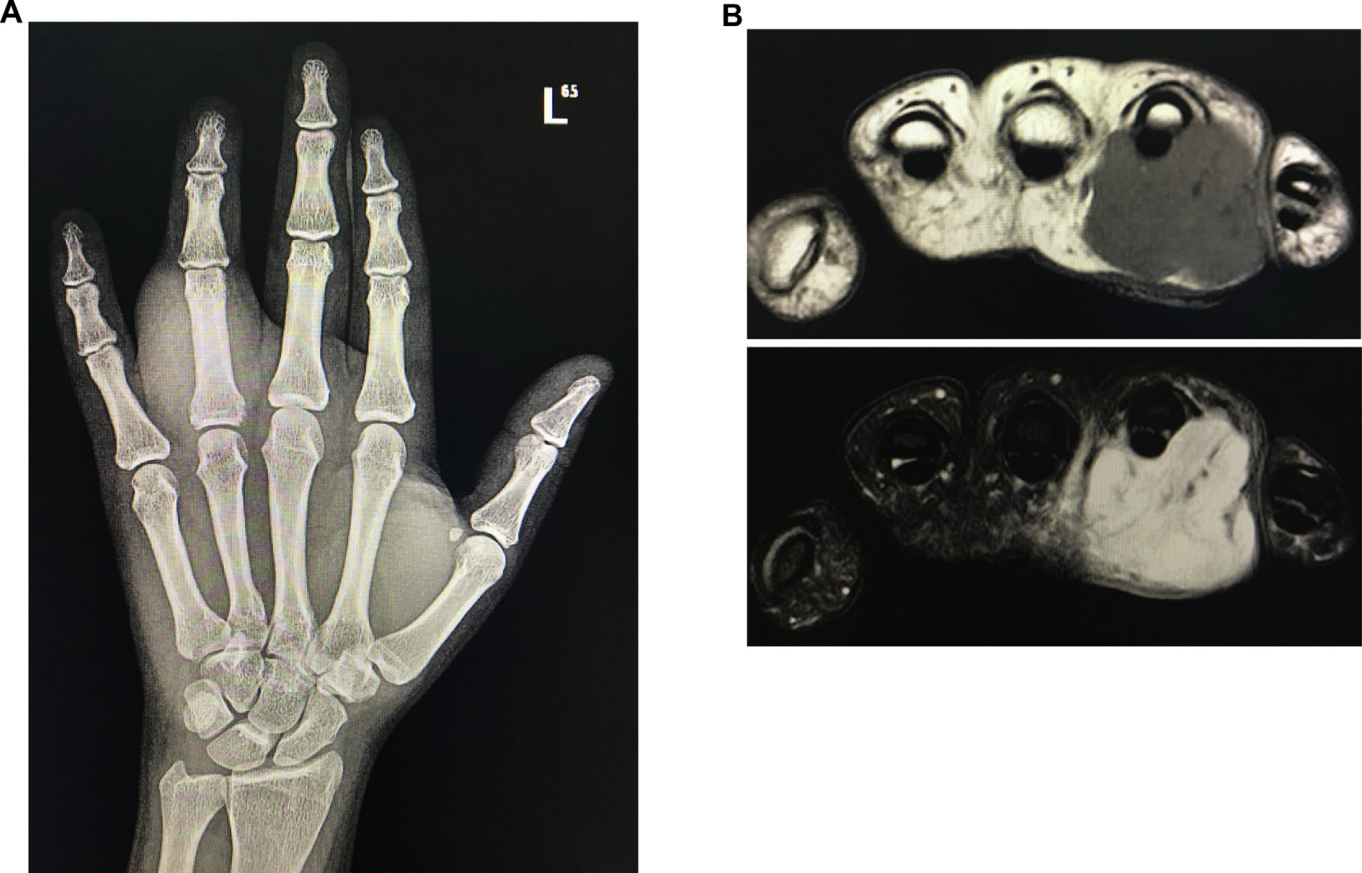
**Pre-operative radiological investigation Fibro-osseous pseudotumor mass. A.** It shows hand x-ray with extensive soft tissue swelling around the proximal phalanx of ring finger. **B.** It shows axial views of both T1-weighted and T2-weighted MRI imaging. Notice the extensive nature of the mass with involvement of neurovascular bundles.

Radiological evaluation of the involved hand showed a soft tissue swelling with no evidence of bone involvement (Fig. 2). Further magnetic resonant (MRI) evaluation showed a mass on the volar aspect of the ring finger encasing about 50% of the flexor tendons of that digit with low signal intensity on T1 and high signal intensity on T2 evaluation with strong enhancement in post contrast evaluation. Assessment of neurovascular structures showed partial abutment of the radial sided bundle together with complete encirclement of the ulnar sided neurovascular bundle. The surrounding bone was free of any masses and associated mass effect.

**Fig. 3:**
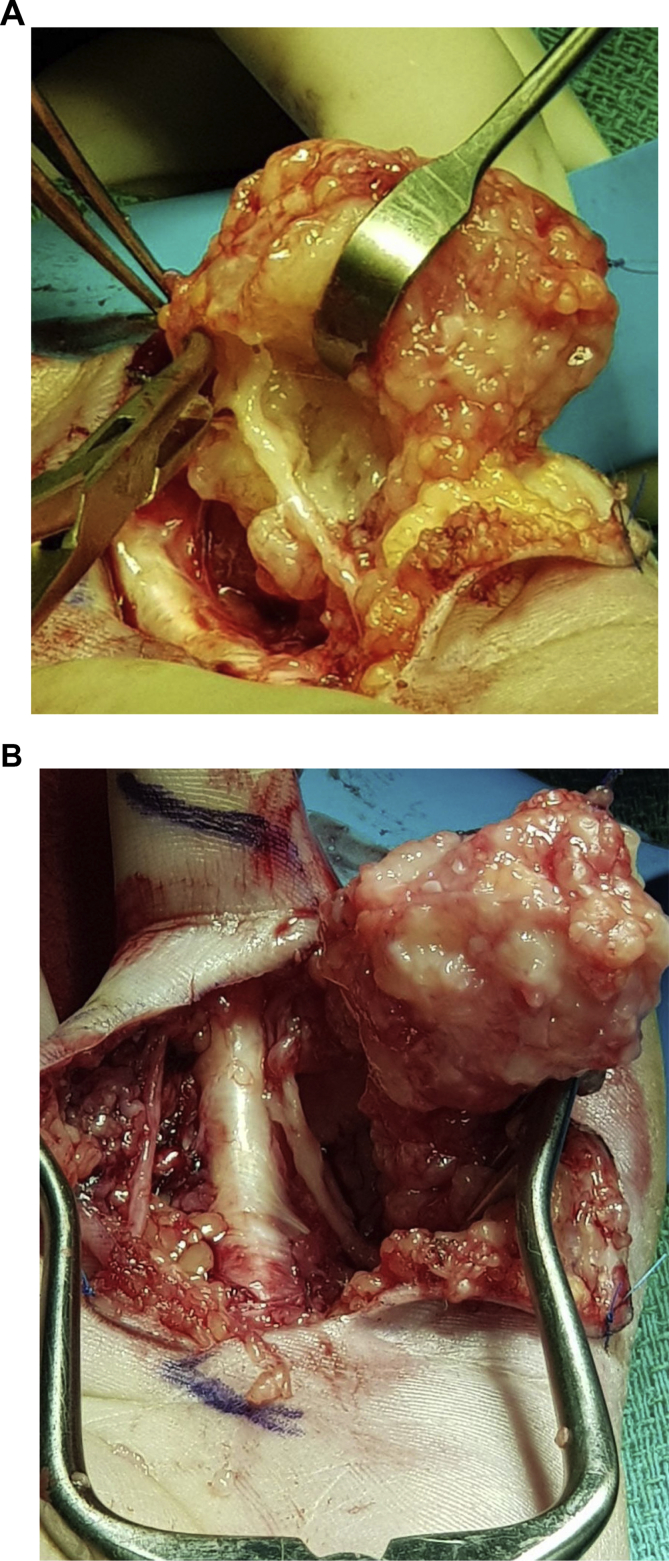
**Intra-operative Fibro-osseous pseudotumor mass dissection. A.** It shows the mass after being dissected freely from the ulnar neurovascular bundle. Notice a remaining distal part of the mass involving the bundle prior to completing the mass dissection. **B**. It shows the mass after being dissected from surrounding vital structures with intact both radial and ulnar neurovascular bundles.

**Fig. 4:**
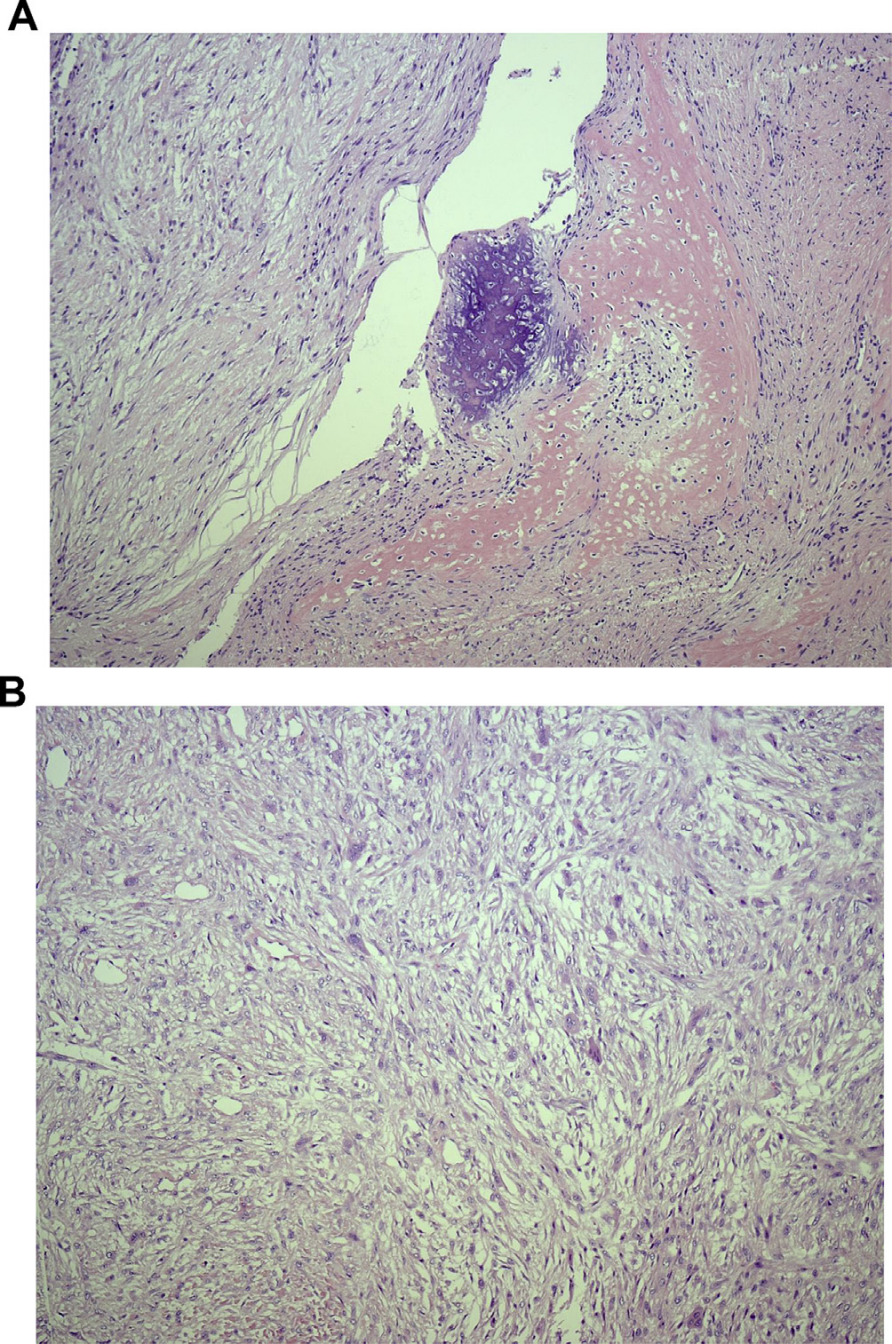
**Histologic Findings. A.** It shows Fibrous and osseous areas that were present throughout the lesion confirming the diagnosis of Fibro-osseous pseudotumor of the digits (FOPD). **B.** It shows atypical cells with multi-nucleated giant cells.

**Fig. 5:**
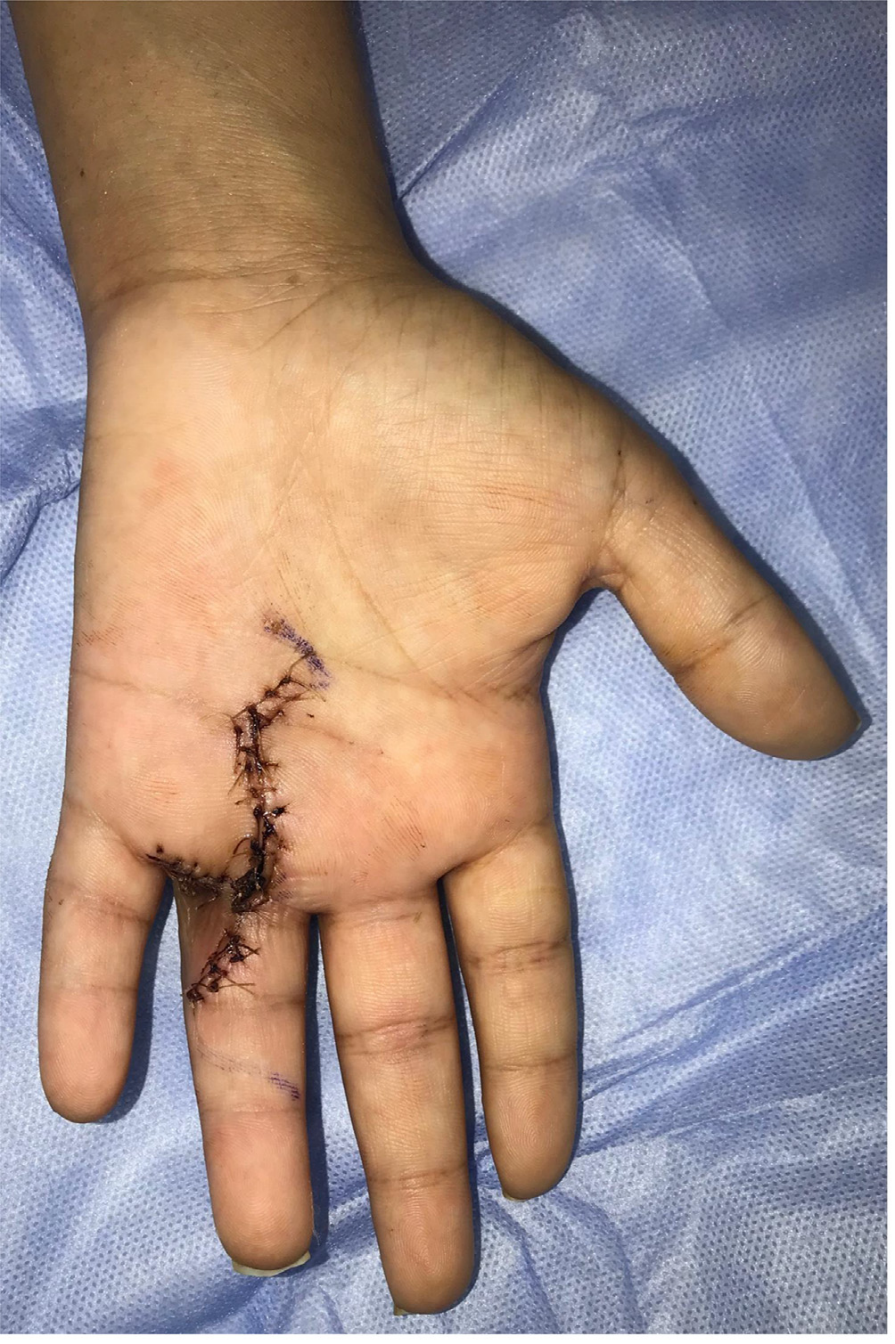
**Post-operative view.** It shows the patients hand after being evaluated at three weeks follow up with no complications or wound dehiscence.

The patient was taken to the OR for exploration and mass excision by the senior author. Possible risks associated with such intervention were explained. Intra-operatively, bruner type incision was designed together with island of skin involved in the mass. Exploration revealed extensive subcutaneous mass with fibro-fatty consistency with extensive fascia like extension to the surrounding soft tissue. The mass was encircling the ulnar neurovascular bundle with mass abutment over the radial bundle as seen in pre-op assessment. The mass was dissected freely from its attachment to those bundles preserving both radial and ulnar structures. The mass was then excised en-bloc having a dimension of 3.5 × 4x2.5 cm (Fig. 3). Histological assessment showed a lesion with fasciitis like features, myofibroblastic proliferation and scattered foci of osteoid formation that was positive for Alpha-Smooth Muscle Actin (ASMA 1A4) immune staining and no evidence of malignancy (Fig. 4). The resected margins were however, positive for residual lesion with difficulty in obtaining negative margins due to the extensive nature of the mass. Post-operatively, the patient had an un-eventual course. She was informed about the need for close follow-ups for both clinical and/or radiological signs of lesion recurrence, pending early surgical intervention (see Fig. 5).

## 4 Discussion

Fibro-osseous pseudotumor (FOPD) of the digit is a rare benign lesion that usually presents clinically as a localized erythematous, painful, subcutaneous fusiform swelling or mass that appears suddenly or gradually in the proximal phalanx of the digits, most commonly in the index finger and very rarely in toes [1,4,5,7]. On gross assessment, it was observed as a well-circumscribed, firm or rubbery lesion with a gray-whitish cut surface and some calcification [1]. It predominantly affects young adults, unlike myositis ossificans, that is more common in women [7]. Although a history of trauma was not considered related to diagnose this lesion, such history can occasionally present in patients with FOPD [1,7,8].

On X-ray, the lesion presents as an ill-defined soft tissue mass with focal calcification, and lack geographic distribution or zoning patterns of myositis ossificans. Focal periosteal thickening adjacent to the mass can also be seen, and in some rare cases, distinct cortical erosions were observed [1,7,9,10]. Magnetic resonant imaging of such tumor was shown to exhibit low to intermediate signal intensity on T1-weighted imaging and variable signal intensities on T2-weighted imaging together with contrast enhancement that were consistent with fibro-proliferative lesions [11]. In our patient pre-operative radiologic imaging was of great help anticipating the nature of mass extensive extension and involvement of neurovascular bundles. Meticulous dissection and handling helped in optimizing the surgical outcome with no residual effect on any vital structures.

Histologically the lesion appears multi-nodular with irregular margins. It consists of a mixture of typical and atypical fibroblasts, osteoblasts and trabeculae of bone with variable degrees of maturations. The proliferation of fibroblasts was either loosely disseminated within a richly mucoid matrix or forming a netlike pattern associated with collagen and islands of loosely cellular tissue. Fibroblasts were seen with abundant amphophilic cytoplasm with indistinct borders and large, slightly pleomorphic nuclei, distinct nuclear membranes, and occasionally small nucleoli. Fibroblast transition to osteoblasts and osteocytes is also a common feature seen under the microscope, as well as the transformation of collagen to osteoid and bone. Osteoblasts however show no cellular atypia, and osteoclasts and bone marrow elements are rarely seen. Multinucleated giant cells are also present. Signs of inflammation were usually minimal and consist of small aggregates of lymphocytes and occasional plasma cells [1,7]. Immune stating of such lesions were found to have focal positivity for alpha smooth muscle (ASMA), S100 and CD 34 markers [8]. Histological assessment of the excised mass in this patient showed similar finding with scattered osteoid formation, myofibroblast proliferation and positivity for ASMA immune-staining.

A diagnostic algorithm was suggested when evaluating such lesions. The first step was to assess the stromal component of the lesion for any evidence of malignancy including; nuclear atypia, pleomorphism, mitotic activity and growth pattern. If malignant evidence was found, then the differentials would include osteosarcoma and other differentiated sarcomas with heterologous differentiation. However, if no evidence of malignancy was found and the stroma was fibroblastic, then the second step was to search for maturation and osteoblastic rimming in the osseous component. Mature osteoid and prominent osteoblastic rimming, would narrow the differentials to fibro-osseous pseudotumor and myositis ossificans. The third step was to look at the location and zoning pattern. If such lesion was present distally and superficial in location with the absence of zonation pattern, this will favor the diagnosis of fibro-osseous pseudotumor. Conversely, if it presented as a proximal deep lesion and zonation patterns were present, these findings would make myositis ossificans a more favorable diagnosis [8].

The major differential diagnoses include Myositis ossificans, extraskeletal osteosarcoma, bizarre parosteal osteochondromatous proliferation and subungal exostosis.

Myositis ossificans is the most commonly confused differential since it shares many histological and clinical features with FOPD. Some authors even suggested that FPOD was the cutaneous counterpart of myositis ossificans [8,12]. Myositis ossificans was more commonly seen in young males and often preceded with trauma [7]. Such defect is also usually found deeper in the soft tissue and associated with more cartilage intermediates [4]. The most reliable way to diagnose Myositis ossifican when in doubt, was through its typical zonation pattern found histopathologically with immature cellular elements in the center and mature bone at the periphery [13]. Radiologic assessment with CT scan is usually sensitive in detecting ossification patterns with central fatty metaplasia, however, MRI assessments are usually superior in showing zonation patterns before ossification appears [14]. The other common differential diagnosis was extra-skeletal osteosarcoma. One of the main defining feature, yet not specific, was the age of the patient at presentation. FOPD usually affects young adults between the ages of 20 and 30, conversely, extra-skeletal osteosarcoma is rarely seen in patients younger than 35 [13]. Extra-skeletal osteosarcoma is also seen more in males and rarely affects the digits. Histologically, it was characterized by its destructive stromal invasion, cytological atypia and the presence of neoplastic osteoid [7,8]. Another common differential is bizarre parosteal osteochondromatous proliferation (BPOP), which like FOPD, arises more proximally in the digits of the hand than the feet. Nonetheless, BPOP is different in the sense that it is typically single, small and usually painless [4,13,15]. It can be distinguished radiologically by its attachment to bone by a stalk, and by pathologic demonstration of a cartilaginous cap. FOPD on the other hand was considered as an extra-osseous lesion, which develops through intramembranous ossification and usually lacks a cartilage intermediate. Finally, BPOP has about a 50% recurrence rate, unlike FOPD, which nearly never recurs when completely excised [13]. Subungal exostosis can also be considered among the differential diagnosis. This lesion is usually located in the distal tip of the digits but can in some cases occur proximally. Subungal exostosis can also be distinguished from FOPD by its connection to the underlying bone through radiological assessment and by the presence of a cartilaginous cap histologically [4,8].

Early diagnosis and treatment of fibro-osseous pseudotumor has shown to be very crucial optimizing the overall outcome. One of the main factors to consider despite no reported cases of malignant transformation to date, is the existing evidence of a definite link between long-standing chronic inflammatory and neoplastic transformation, thus making it necessary to intervene as soon as possible [16]. Additionally, due to the location of the lesion being more superficial in the dermis, it makes it prone for ulceration and secondary infections [8]. Diagnostic ambiguity was clearly seen when dealing with such lesion in which combined clinical and pathological features usually help in narrowing the differential diagnosis and direct patient prognosis.

Complete excision of the lesion is the golden standard treatment modality for fibro-osseous pseudotumor of the digit. The prognosis of this lesion, when completely excised, was favorable with a low risk of local recurrence (0–14% in various series) [17] and no evidence of malignant transformation. Incomplete excision however of the tumor was associated with a higher chance of lesion recurrence [1,4,5]. In this case however, complete excision was challenged by the extensive degree of tumor invasion. Close patient follow-ups were recommended in this situation, in which any evidence of tumor recurrence will usually require surgical re-excision and in some situations ray-amputation [1,4]. Patient counseling must be directed toward the nature of the disease but due to its differential, close follow-ups must be assured. This patient was put on a 3-monthly follow-up plan with both clinical and radiological (MRI) evaluation. Any evidence of recurrence will mandate immediate surgical re-excision with possible consideration of ray amputation being the extent of recurrence.

## Ethical approval

This study was approved by the Ethics Committee at the national guards health affairs. The patient was consented for the publication of this work.

## Sources of funding

None.

## Author contribution

Dr. T. A. Jawadi, was involved in the overall manuscript organization.

Dr. F. AlShomer, was involved in data collection and overall manuscript writing.

Dr. M. Almutairi, was involved in abstract and paper collection.

Dr. A. Alqahtani, was involved in patient follow-ups, histology reviewing.

Dr. M. Alfowzan, was involved in the radiology data reporting and figures assembly.

Dr. Al-Meshal O was involved, figure drawing and editing and proof-reading the manuscript.

## Conflicts of interest

The authors report no conflicts of interest. The authors alone are responsible for the content and writing of the paper.

## Research registration unique identifying number (UIN)

Not applicable.

## Guarantor

Feras alshomer.

## Patient consent

The patient consented for the publication of this work.

## Provenance and peer review

Not commissioned, externally peer reviewed.

## Appendix A Supplementary data

SCARE Guideline Checklist SCARE Guideline Checklist
